# P057. Prophylaxis with low-dose methadone in patients affected by daily refractory headache and medication-overuse headache: a prospective cohort study (METACEF study)

**DOI:** 10.1186/1129-2377-16-S1-A118

**Published:** 2015-09-28

**Authors:** Chiara Lupi, Chiara Pracucci, Francesco De Cesaris, Eleonora Rossi, Pierangelo Geppetti, Silvia Benemei, Valentina Galli, Brunella Occupati, Viola Mazzucco, Guido Mannaioni

**Affiliations:** Headache Centre, Careggi University Hospital, University of Florence, Florence, Italy; Toxicology Unit, Careggi University Hospital, Florence, Italy

## Objective

To evaluate the effectiveness, safety, and tolerability of a 12-month treatment with low doses of methadone (MT) (mean MT dosage 12.3 mg ± SD 7.3) as prophylaxis in patients affected by daily refractory headache and medication-overuse headache.

## Methods

Prospective cohort study.

## Results

Since May 3^rd^, 2012 up to January 8^th^, 2015, we enrolled 24 patients (18 females, 6 males; average age, 48 years) who were considered eligible to be treated with methadone. Nine patients dropped out because of adverse drug reactions (n=4, mean time of drop-out 7 days) or treatment ineffectiveness (n=5, mean time of drop-out 6 months). Six patients completed the 12-month treatment. After 1-year follow-up they still reported daily headache, however, they showed an impressive decrease of analgesic and/or antimigraine drug consumption (from 147.7 medications per month ± SD 124 to 8.5 medications per month ± SD 6.1) and a significant decrease of visual analogic scale (VAS) pain intensity (from 5.8 ± SD 2.6 to 2.8 ± SD 2.1). These patients were treated with daily methadone doses ranging from 5 mg to 60 mg; methadone dosages were safe and well tolerated.

## Conclusions

In patients affected by daily refractory headache and medication-overuse headache, who are exposed to the risk of serious side effects due to prolonged analgesic and/or antimigraine treatment, prophylaxis with low-dose methadone therapy seems to represent an effective therapeutic option.

Written informed consent to publication was obtained from the patient(s).

